# Mitochondrial uncoupling protein 2: a central player in pancreatic disease pathophysiology

**DOI:** 10.1186/s10020-024-01027-y

**Published:** 2024-12-20

**Authors:** Kunpeng Wang, Lilong Zhang, Beiying Deng, Kailiang Zhao, Chen Chen, Weixing Wang

**Affiliations:** 1 Department of General Surgery, Renmin Hospital of Wuhan University, Wuhan, China; 2 General Surgery Laboratory, Renmin Hospital of Wuhan University, Wuhan, China; 3 Department of Gastroenterology, Renmin Hospital of Wuhan University, Wuhan, China

**Keywords:** Mitochondrial uncoupling protein 2, Pancreatic diseases, Pancreatitis, Pancreatic cancer, Diabetes mellitus, Reactive oxygen species

## Abstract

Pancreatic diseases pose considerable health challenges due to their complex etiology and limited therapeutic options. Mitochondrial uncoupling protein 2 (UCP2), highly expressed in pancreatic tissue, participates in numerous physiological processes and signaling pathways, indicating its potential relevance in these diseases. Despite this, UCP2’s role in acute pancreatitis (AP) remains underexplored, and its functions in chronic pancreatitis (CP) and pancreatic steatosis are largely unknown. Additionally, the mechanisms connecting various pancreatic diseases are intricate and not yet fully elucidated. Given UCP2’s diverse functionality, broad expression in pancreatic tissue, and the distinct pathophysiological features of pancreatic diseases, this review offers a comprehensive analysis of current findings on UCP2’s involvement in these conditions. We discuss recent insights into UCP2’s complex regulatory mechanisms, propose that UCP2 may serve as a central regulatory factor in pancreatic disease progression, and hypothesize that UCP2 dysfunction could significantly contribute to disease pathogenesis. Understanding UCP2’s role and mechanisms in pancreatic diseases may pave the way for innovative therapeutic and diagnostic approaches.

**Graphical Abstracts:**

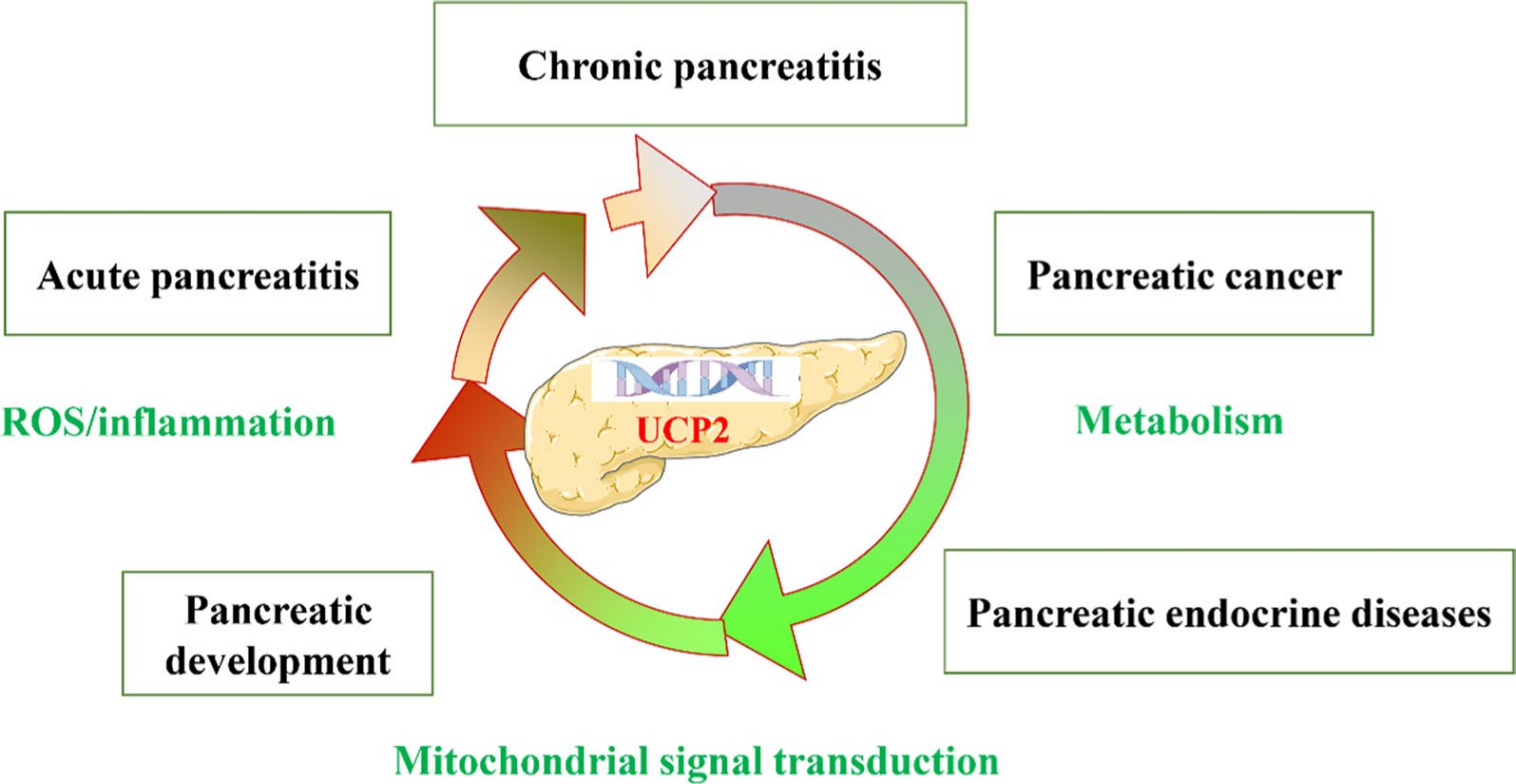

## Introduction

Mitochondria were traditionally regarded as the primary energy centers of eukaryotic cells (Zheng et al. 2023). However, a deeper understanding of mitochondria has revealed increasing evidence that they function as multifunctional, dynamic organelles engaged in genetic information processing, energy conversion, biosynthesis, and signal transduction. These organelles are essential components of the mitochondrial information processing system (MIPS) and play a crucial role in biological regulation through three primary steps: sensing, integration, and signal transduction (Picard and Shirihai 2022). Uncoupling proteins (UCPs), a class of mitochondrial carriers, primarily regulate reactive oxygen species (ROS) production during mitochondrial oxidative phosphorylation and also participate in mitochondria-related signal transduction (Cadenas 2018).

UCP2 is the most widely distributed uncoupling protein in the human body, with broad expression across the brain, liver, pancreas, muscle, and immune cells, where it plays a pivotal role in energy homeostasis and the regulation of ROS (Luby and Alves-Guerra 2022). Its critical functions in oxidative stress management and metabolic regulation, particularly its impact on insulin secretion and glucose and lipid metabolism, have drawn significant research interest (Diano and Horvath 2012). Importantly, UCP2 exhibits over 95% homology between humans and mice, a much higher similarity compared to other UCPs (Caggiano and Taniguchi 2024). Further underscoring its research relevance.

The pancreas, a digestive gland with both endocrine and exocrine functions, plays a vital role in regulating various metabolic processes. Pancreatic dysfunction can result in conditions such as pancreatitis, diabetes, and pancreatic cancer (Guillaumond et al. 2014; Schlünder et al. 2024). Rising incidence and prevalence of pancreatic diseases contribute to a substantial healthcare burden (Ouyang et al. 2020; Chen et al. 2020). UCP2 has attracted significant research interest due to its extensive expression in pancreatic tissue and its crucial roles in oxidative stress regulation and metabolic processes (Zhang et al. 2001; Galetti et al. 2009). Studies suggest that UCP2 influences the proliferation of pancreatic islet α and β cells, as well as the secretion of insulin and glucagon, thereby affecting glucose and lipid metabolism (Luo et al. 2022). Additionally, UCP2 may be involved in pancreatic development via the ROS-AKT signaling pathway (Broche et al. 2018). In models of acute pancreatitis (AP), UCP2 knockdown inhibits the proliferation of pancreatic stellate cells (Muller et al. 2016), and modulates macrophage redox responses, impacting the progression of KRAS-associated pancreatic cancer (Raho et al. 2020).

The role of UCP2 in acinar cell injury and macrophage regulation during AP remains unclear, and studies on UCP2 in chronic pancreatitis (CP) are limited. It is also unknown whether UCP2 influences pancreatic fat infiltration or fatty pancreas development, and by what mechanisms this may occur (Petrov 2023). Additionally, the mechanisms underlying recurrent AP, fibrosis in CP, and progression to pancreatic cancer are poorly understood, with few effective clinical targets available. The impact of pancreatitis episodes on glucose regulation and the development of diabetes also requires further investigation. Given UCP2’s diverse functions, widespread expression in pancreatic tissue, and the interconnected pathophysiology of pancreatic diseases, this review examines current findings on UCP2’s regulatory role, proposing that UCP2 dysfunction may play a central role in pancreatic disease pathogenesis. Understanding UCP2’s mechanisms could offer novel therapeutic and diagnostic insights.

## Regulation of UCP2 in pancreatic diseases

The regulation of UCP2 in pancreatic diseases encompasses several mechanisms, including gene mutations, transcription factors influencing UCP2 expression in pancreatic diseases, and UCP2-related epigenetic modifications. Investigating UCP2 regulation is essential for understanding its role in pancreatic diseases and underscores its potential as a central therapeutic target.

### Mutations of UCP2 in pancreatic diseases

Genetic polymorphism, defined as the presence of two or more allelic variants of a gene at the same locus with a variation frequency generally exceeding 1%, can affect gene expression and function, leading to biological differences between individuals (Krauss et al. 2005). Genetic polymorphism is a crucial source of biodiversity and serves as the basis for evolution and natural selection. Major types of genetic polymorphisms include single nucleotide polymorphisms (SNPs), insertion/deletion polymorphisms (Indels), repetitive sequence polymorphisms (RSPs), and structural variants (SV) (Hayashi et al. 2021).

The SNPs of UCP2 primarily include the 866G/A polymorphism in the promoter region and the Ala55Val polymorphism in the exon region. The Indels mainly involve the insertion of a 45 bp sequence in exon 8 of the 3′ untranslated region of the UCP2 gene (Jia et al. 2009; Donadelli et al. 2014). The relative mean mutation frequencies of 866G/A and Ala55Val were similar, at approximately 37% and 39.6%, respectively (Dalgaard 2011). Additionally, the 866G/A and Ala55Val polymorphisms may have a combinatorial effect; for example, individuals carrying both the 866G/A and Val55 alleles may exhibit higher UCP2 activity and stronger antioxidant capacity (Nicoletti et al. 2017).

The distribution and frequency of these UCP2 gene polymorphisms may vary among different populations and can affect susceptibility to pancreas-related diseases, such as insulin secretion and type 2 diabetes mellitus, differently in various individuals and genders (Andersen et al. 2013; Souza et al. 2013). By studying these polymorphisms and their functional significance, the role of UCP2 in pancreatic diseases can be better understood. Two studies have comprehensively summarized the impact of UCP2 gene polymorphisms on metabolic diseases (Jia et al. 2009; Donadelli et al. 2014). We have built upon these studies to summarize and update our understanding of the role of UCP2 gene polymorphisms in pancreatic diseases in recent years (Table 1).

**Table 1:** Summary and update of the role of UCP2 gene polymorphisms in pancreatic diseases in recent years

Years	Race	UCP2 Genetic polymorphism	Biological effect	Refs.
2006	Caucasians	866G/A	Type 2 diabetes susceptibility	Gable et al. (2006)
2002	Austrian Caucasians	866G/A	Inhibits insulin secretion	Krempler et al. (2002)
2004	Japanese	866G/A	Inhibits insulin secretion	Sasahara et al. (2004)
2004	Italian Caucasian	866G/A	Increased risk of type 2 diabetes	D'Adamo et al. (2004)
2005	Americans	Ala55Val	Increased risk of type 2 diabetes	Yu et al. (2005)
2010	Northern Indians	866G/A	Increased risk of Hyperinsulinemia	Srivastava et al. (2010)
2013	Danes	866G/A	Increased risk of type 2 diabetes	Andersen et al. (2013)
2011	Asian descent	Ala55Val	Increased risk of type 2 diabetes	Xu et al. (2011)
2009	European American women	Ala55Val	Increased risk of type 2 diabetes	Willig et al. (2009)
2011	Asian Indians	Ala55Val and −55C/T	Decreased risk of type 2 diabetes	Vimaleswaran et al. (2011)
2008	Koreans	UCP2 −5331G > A and UCP3 −2078C > T	Increased risk of type 2 diabetes	Lee et al. (2008)
2008	patients form Necker-Enfants Malades Hospital	Ucp2 variants (G174D and A268G)	Promotes insulin secretion	González-Barroso et al. (2008)
2023	Kashmiri population of Northern India	866G/A	Increased risk of type 2 diabetes	Din et al. (2023)
2021	Asians	866G/A	Decreased risk of type 2 diabetes	Huang et al. (2021)
2021	Asians	Ala55Val	Increased risk of type 2 diabetes	Huang et al. (2021)
2021	Russians	Ucp2 T/T variant	Increased risk of type 2 diabetes	Lapik et al. (2021)
2021	North-west of Iran	45 bp I/D polymorphism in 3'UTR of UCP2	Increased risk of type 2 diabetes	Rezapour et al. (2021)
2021	Asians	866G/A	Increased risk of type 2 diabetes	Xu et al. (2021a)
2020	Northern Chinese population	866G/A	Increased risk of type 2 diabetes	Hou et al. (2020)
2019	South Indian population	866G/A	Increased risk of type 2 diabetes	Gomathi et al. (2019)
2013	Asians	UCP2 Ala55Val and UCP3 −55C/T	Increased risk of type 2 diabetes	Souza et al. (2013)

Of the 21 studies we summarized, 11 focused on the −866G/A polymorphism of UCP2. Except for one study that indicated the −866G/A polymorphism reduces the risk of type 2 diabetes in Asian populations (Huang et al. 2021), the remaining studies showed that the −866G/A polymorphism predisposes individuals to an increased risk of developing type 2 diabetes. Similarly, 6 out of seven studies on the Ala55Val polymorphism associated it with an increased risk of type 2 diabetes, with the single study showing a negative association also based on an Asian population (Vimaleswaran et al. 2011). This may be related to selection bias in the studies. Additionally, one study showed that UCP2 variants (G174D and A268G) promoted insulin secretion (Lee et al. 2008), while another indicated that the UCP2 T/T variant increased the risk of type 2 diabetes (Lapik et al. 2021). Collectively, we conclude that genetic polymorphisms in UCP2 increase the risk of type 2 diabetes mellitus.

### Transcription factor of UCP2 in Pancreatic Diseases

**Fig. 1:**
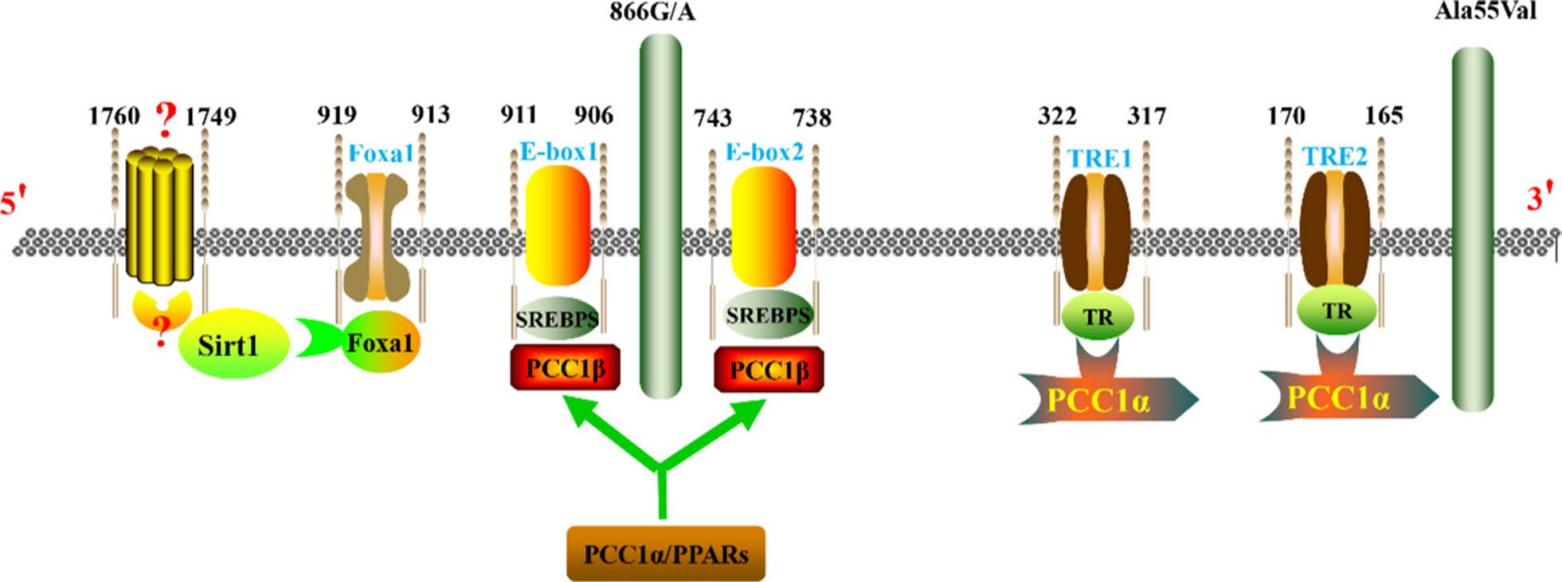
The human UCP2 gene transcription and mutations

The transcriptional regulation of the UCP2 gene encompasses various mechanisms, such as transcription factors, cis-acting elements, epigenetic modifications, and environmental influences. These intricate regulatory mechanisms precisely control UCP2 gene expression under diverse physiological and pathological conditions, thereby fully elucidating the role of UCP2 in pancreatic diseases. The mouse and human UCP2 genes are located on chromosomes 7 and 11, respectively. Both genes comprise eight exons (six coding and two non-coding) and seven introns (Donadelli et al. 2014). The human UCP2 gene transcription and mutations are detailed schematically in Fig. 1. Transcription factors that bind to the UCP2 promoter include forkhead box protein O1(Foxa1), silent mating type information regulation 2 homolog-1, (SIRT1), sterol regulatory element binding protein isoforms (SREBP), thyroid hormone response elements (TRE), and helix-loop-helix protein binding sites (E-box).

Initial interest in the role of Foxa1 in pancreatic disease arose from observations of hypoglycemia and abnormal changes in glucose metabolism in Foxa1 knockout mice (Shih et al. 1999). Subsequent studies in β-cells revealed reduced ATP synthesis following Foxa1 knockdown, accompanied by increased expression of UCP2. Chromatin immunoprecipitation assays further confirmed UCP2 as a direct transcriptional target of Foxa1 in vivo (Vatamaniuk et al. 2006). More importantly, Foxa1 has been shown to repress UCP2 gene transcription by binding to the − 919 to − 913 elements (Song et al. 2014). Controversially, Foxa1 is suggested to bind to the Ucp2 promoter at a preferred site located between − 1760 and − 1749 bp relative to the gene’s transcription start site, yet conclusive direct evidence is lacking (Donadelli et al. 2014). Additionally, a recent study demonstrated that silencing Foxa1 promotes UCP2 expression (Bao et al. 2022).

It was reported that peroxisome proliferator-activated receptor-γ coactivator-1 α (PGC-1α) promotes thyroid hormone-mediated transcriptional activation of the UCP2 gene in INS-1E cells (Oberkofler et al. 2009). Hannes Oberkofler et al. (Oberkofler et al. 2006) identified two TREs at positions − 322/− 317 (TRE1) and − 170/− 165 (TRE2). Mutations in TRE1 or TRE2 attenuated the stimulatory effects of thyroid hormone treatment. Additionally, two E-box motifs at positions − 911/− 906 (E1) and − 743/− 738 (E2) regulate UCP2 gene expression through SREBP-1a, SREBP-1c, and SREBP-2. Mutational analyses indicate that the presence of E1 or E2 alone is sufficient for the nuclear active SREBP-mediated activation of UCP2 gene transcription (Oberkofler et al. 2006). Moreover, miR-23a induces the expression of PGC-1α and also enhances the expression levels of UCP2 (Wang et al. 2015).

Elevated levels of long-chain fatty acids stimulate UCP2 expression, primarily mediated via peroxisome proliferator-activated receptors (PPARs) and SREBPs (Zhou et al. 2016; Chen et al. 2014). The PPAR family includes three principal genes: PPAR-α, PPAR-β, and PPAR-γ, while SREBP exists in three main isoforms: SREBP-1a, SREBP-1c, and SREBP-2 (Shimano 2009). Several studies have confirmed the potentially critical role of PPARs in pancreatic diseases, including protecting pancreatic islet β-cells from metabolic stress, enhancing insulin secretion, and mitigating lipotoxicity (Chen et al. 2015; Hogh et al. 2014; Jiang et al. 2010). Unlike Foxa1, SREBP, TRE, and E-box, which possess binding sites on the UCP2 promoter, no binding sites for PPAR have been identified within or near the Ucp2 gene. Therefore, the regulation of UCP2 by PPARs appears to be indirect (Donadelli et al. 2014). However, it has been documented that PPARs bind the direct repeat sequence 5′-AGGTCA-3′ as a specialized heterodimer with the retinoid X-like receptor (RXR) (IJpenberg et al. 1997; Gearing et al. 1993). Additionally, PPARs require a double E-box motif in their proximal promoter for their biological functions. Further investigation is necessary to confirm the regulatory role of PPARs in UCP2 gene transcription in future studies (Medvedev et al. 2001).

In β-cells, SIRT1 inhibits UCP2 transcription by directly binding to its promoter, thereby affecting insulin secretion (Bordone et al. 2006; Moynihan et al. 2005). SIRT1 also interacts with various transcription factors of UCP2. For example, it suppresses PPARγ, thereby regulating white adipose tissue function (Zu et al. 2020). The SIRT1-Ppargc1a-Ucp2 pathway is associated with insulin resistance and obesity (Kettunen et al. 2024). Additionally, SIRT1 modulates Foxa1, influencing cellular metabolic levels possibly due to its proximity to the Foxa1 binding site on the UCP2 promoter (Bordone et al. 2006). Moreover, SIRT1 synergizes with peroxisome proliferator-activated receptor coactivator PGC-1α (Xu et al. 2021b).

TGFβ signaling negatively regulates UCP2, as demonstrated in tumor cells where low malignancy levels suppress gene transcription by recruiting TGFβ-induced SMAD4 to six repressive SMAD-binding elements (RSBEs, − 100 to − 354) on the UCP2 promoter (Sayeed et al. 2010). Conversely, highly malignant tumor cells promote UCP2 expression. Additionally, glutamine induces UCP2 protein translation in a concentration-dependent manner. Insufficient glutamine inhibits UCP2 protein translation due to a short upstream open reading frame (uORF) consisting of 36 amino acids in the 5' untranslated region. In the presence of glutamine, the inhibitory effect of uORF on translation is alleviated (Hurtaud et al. 2007).

### Epigenetic mechanisms of UCP2 in pancreatic diseases

The epigenetic regulation of UCP2 involves DNA methylation, histone modifications, non-coding RNAs (ncRNAs), and chromatin remodeling. These mechanisms are not independent; rather, they frequently interact synergistically, with transcription factors also contributing to their regulation. Consequently, the epigenetic regulation of UCP2 must be understood holistically.

**Fig. 2:**
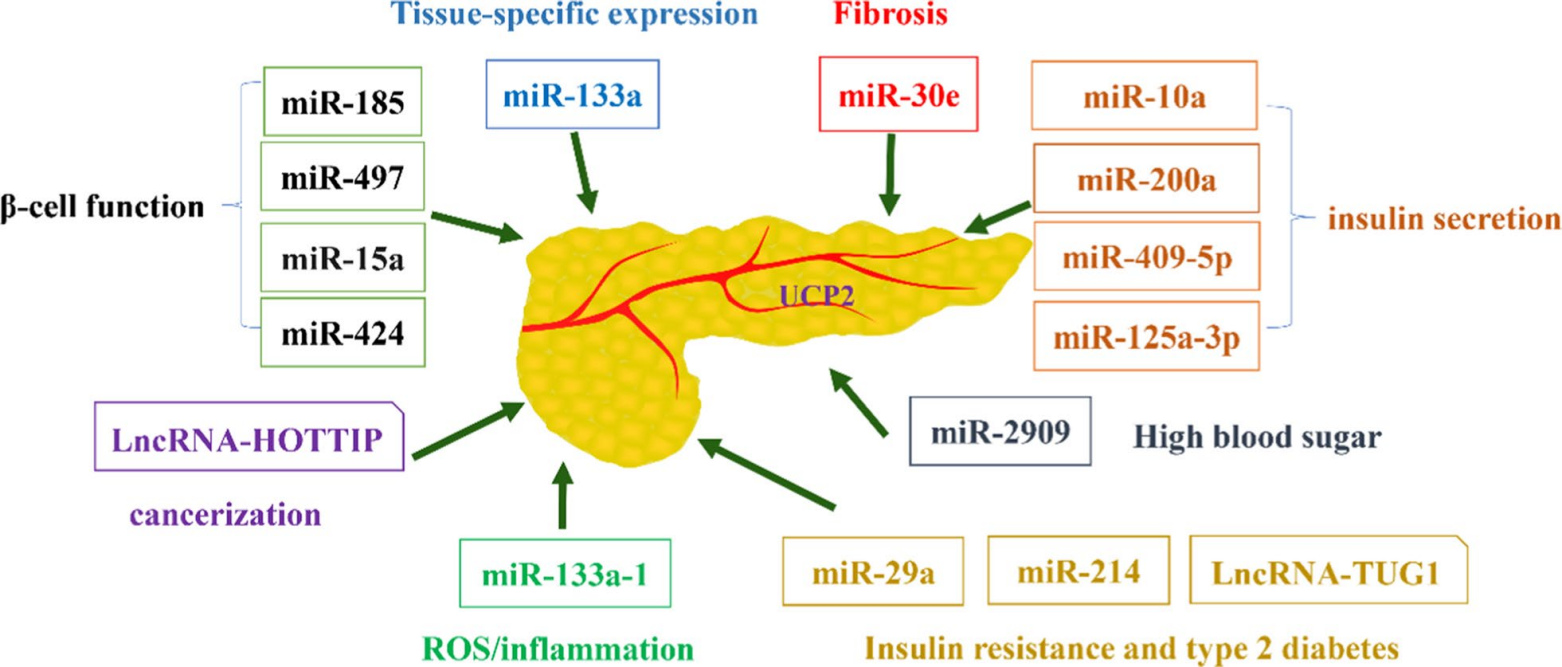
The relationship between ncRNAs and UCP2

AMPK has been shown to enhance histone acetylation by phosphorylating DNMT1, RBBP7, and HAT1, which in turn reduces DNA methylation and chromatin remodeling at the UCP2 promoter (Marin et al. 2017). UCP2 also regulates acetyl-CoA levels, histone acetylation, and chromatin remodeling within the metabolic microenvironment (Rigaud et al. 2022). ncRNAs play a crucial role in the epigenetic regulation of UCP2 and have potential as biomarkers for diagnosing and prognosing pancreatic diseases (Liu et al. 2019). Specific microRNAs (miRNAs) and long non-coding RNAs (lncRNAs) can serve as non-invasive biomarkers for the early detection and monitoring of CP, diabetes, and other pancreatic disorders. Furthermore, ncRNAs are vital regulators of pancreatic diseases, influencing inflammation, fibrosis, insulin secretion, and cell survival (Xiong et al. 2019).In this section, we summarize the ncRNAs involved in the epigenetic regulation of UCP2 (Fig. 2).

MiR-133a plays a role in targeting and regulating the tissue-specific expression of UCP2 (Chen et al. 2009). However, miRNAs with analogous roles have not been identified in pancreatic tissue. miR-15a inhibition of endogenous UCP-2 protein levels is a critical regulator of β-cell function and insulin biosynthesis (Sun et al. 2011). Conversely, miR-15a, miR-424, miR-497, and miR-185 directly target the 3'UTR of UCP2 mRNA to suppress its expression, forming a regulatory network that influences β-cell function (Lang et al. 2018). Some researchers have explored the potential association between hypothalamic miRNA expression profiles and insulin responsiveness, identifying 34 up-regulated miRNAs and 4 down-regulated miRNAs. They specifically investigated the expression of miR-10a, miR-200a, miR-409-5p, and miR-125a-3p (Benoit et al. 2013). Another study highlighted the involvement of miR-2909 in regulating UCP2 expression, particularly in hyperglycemic conditions (Kaul et al. 2015). miR-29a impacts glucose and lipid metabolism, presenting as a potential target for managing insulin resistance and type 2 diabetes (Wu et al. 2018). Additionally, miR-214 and lncRNA TUG1 regulate UCP2 expression levels and play pivotal roles in insulin resistance and type 2 diabetes (Wei et al. 2022; Yang et al. 2019).

MiR-133a-1 inhibits the activation of NLRP3 inflammasomes by suppressing UCP2 (Bandyopadhyay et al. 2013). Interestingly, miR-133a-3p exhibits a positive correlation with UCP2 expression and a negative correlation with IL-18 (Bandyopadhyay et al. 2013). Additionally, the miR-133a/UCP2 signaling axis regulates downstream inflammation, oxidative stress, and energy metabolism (Jin et al. 2017). These findings suggest that miR-133a may hold potential value in the pathogenesis of AP, although no studies have yet been reported in this area. Notably, the miR-30e/UCP2 axis demonstrates significant relevance in renal fibrosis, implying potential applicability in fibrosis-characterized CP (Jiang et al. 2013). Furthermore, lncRNA HOTTIP regulates UCP2 to promote PDAC progression (Wong et al. 2020). However, there are no reports of circRNA regulating UCP2, with circRNA UCP2 involvement only documented in lung cancer (Du et al. 2023).

## Pathological implications of UCP2 in pancreatic diseases

Typically, the primary function of UCP2 is to regulate cellular energy transduction and mitochondrial ROS generation. This makes it an attractive therapeutic target for addressing metabolic imbalance in pancreatic cancer and oxidative damage in pancreatitis (Caggiano and Taniguchi 2024; Jin et al. 2023). As research on UCP2 progresses, a clue to this discrepancy may differ in other organs, the unique role of UCP2 in the pancreas was demonstrated increasingly, with its impact on pancreatic biological functions gradually being uncovered. Significant changes in insulin and blood glucose levels have been observed in UCP2 knockout mice (González-Barroso et al. 2008; Zhou et al. 2009). Detailed studies have elucidated the biological mechanisms by which UCP2 regulates the functions of pancreatic alpha and beta cells as well as blood glucose control (Gomathi et al. 2019; Allister et al. 2013; Mizusawa et al. 2022). Additionally, the function of UCP2 in the development, transplantation, and autoimmune regulation of the pancreas, particularly the islets, has been confirmed (Zhang et al. 2011; Pi et al. 2009; Emre et al. 2007). Given the significant role of UCP2 in the pancreas and pancreatic diseases, this review focuses on the recent research progress regarding the involvement of UCP2 in pancreatic development, pancreatitis, pancreatic endocrine diseases, and pancreatic cancer.

### Pancreatic development

**Fig. 3:**
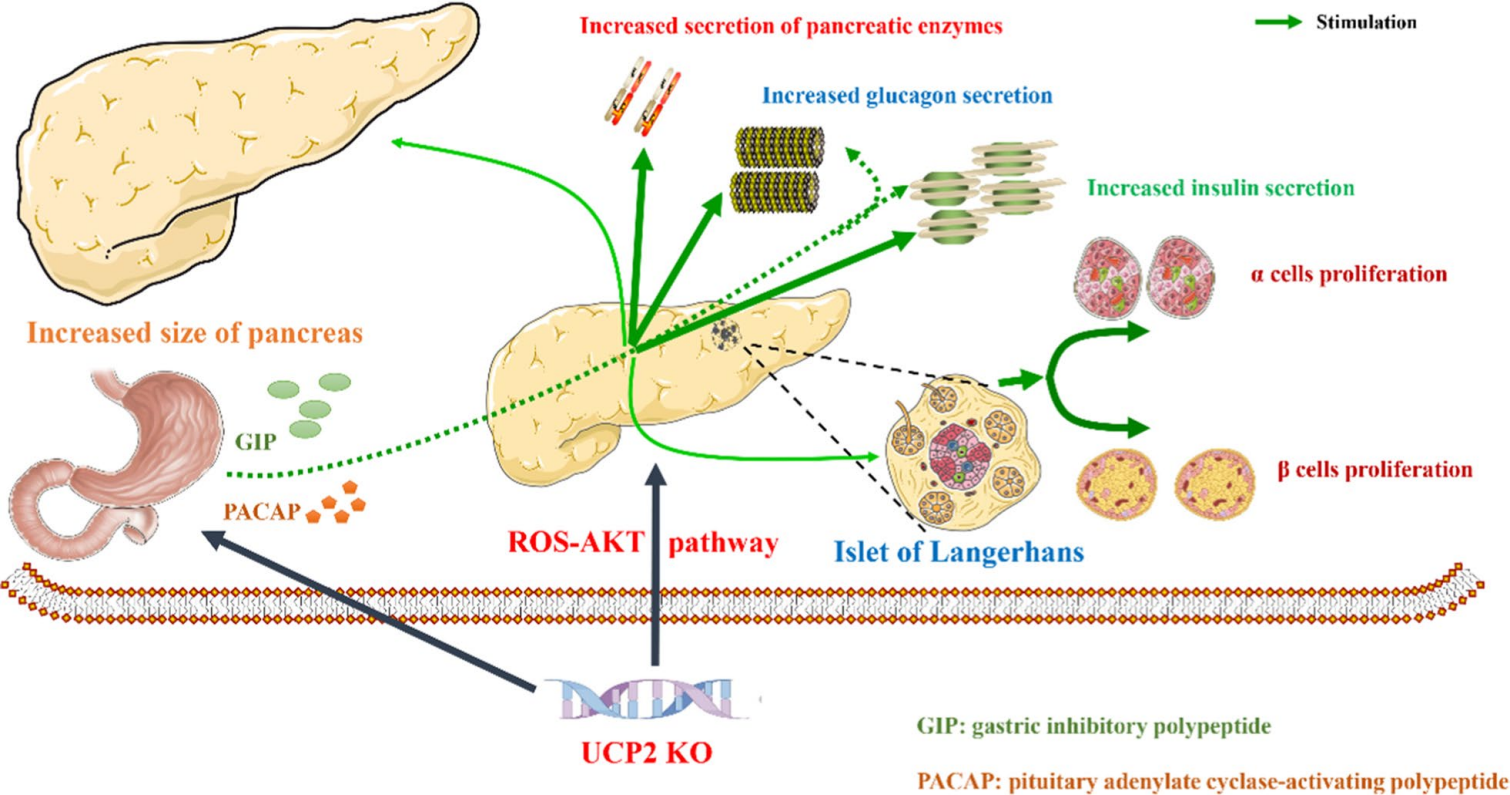
The potential roles of UCP2 in pancreatic development

This section examines the physiological functions of UCP2 in pancreatic development, islet transplantation, and the two major islet cell types (alpha and beta cells), along with its role in regulating somatostatin, pancreatic polypeptides, and ghrelin. Figure 3 summarizes the potential roles of UCP2.

Over the past decade, the effect of mitochondrial dysfunction on pancreatic islet development has been extensively investigated. Mutations in the human UCP2 gene are related to congenital hyperinsulinism (González-Barroso et al. 2008). The deletion of UCP2 in mice is associated with increased insulin secretion and elevated proliferation of endocrine cells (Zhang et al. 2001) a phenomenon that is more pronounced in mice on a high-fat diet (Joseph et al. 2002; Lee et al. 2009). To clarify the impact of UCP2 on pancreatic development, Benjamin Broche et al. (Broche et al. 2018) generated UCP2 whole-body knockout mice at various stages (from embryonic day 9.5 to 19.5) to observe the effects of UCP2 deficiency on pancreatic growth and development. Their results indicated that UCP2 is primarily expressed in pancreatic endocrine cells rather than stromal, epithelial, or other cell types. The absence of UCP2 resulted in significantly larger pancreatic volumes in late-stage embryos compared to controls, and the expression levels of insulin, glucagon, and amylase were significantly increased in fetal and neonatal mice compared to the control group. This phenotype may be related to the proliferation of pancreatic progenitor cells and the activation of the ROS-AKT signaling pathway (Broche et al. 2018).

Islet transplantation is an effective method for treating uncontrollable diabetes, such as recurrent hypoglycemia and insulin desensitization. However, the challenge of restoring pancreatic β-cell function after transplantation limits the clinical application of this technique (Rickels and Robertson 2019). Studies indicate that downregulating UCP2 may mitigate brain death post-islet transplantation and enhance the recovery of pancreatic β-cell function. This may be related to the high expression of UCP2 mediating systemic inflammation and pancreatic β-cell apoptosis (Brondani et al. 2017). The exact biological role of UCP2 in pancreatic islet cells remains controversial due to the mutual influence between α-cells and β-cells, making it difficult to distinguish causal from concomitant effects (Diao et al. 2008). The successful construction of islet α- and β-cell-specific UCP2 knockout mouse models has, fortunately, provided a clearer understanding of the physiological functions of UCP2 in pancreatic islet cells (Allister et al. 2013; Hardy et al. 2011).

Similarly, in a β-cell-specific UCP2 overexpression mouse model, increased levels of UCP2 are associated with glucose intolerance, inadequate insulin secretion, and pancreatic β-cell failure in mice (Inoue et al. 2022). Unlike pancreatic β-cells, the function of UCP2 in pancreatic α-cells is likely more comprehensive. This may be due to the significantly higher expression of UCP2 in pancreatic islet α-cells compared to β-cells (Diao et al. 2008).In islet β-cells, UCP2 knockdown primarily regulates blood glucose levels by increasing ROS production and promoting insulin secretion, with minimal effects on mitochondrial membrane potential and ATP production (Lee et al. 2009). In pancreatic islet α-cells, UCP2 not only functions similarly to β-cells in endocrine regulation at low glucose concentrations but also plays an electroactive regulatory role. UCP2 regulates glucagon secretion to maintain blood glucose levels by modulating ATP generation, plasma membrane potential, and ROS levels (Allister et al. 2013; Robson-Doucette et al. 2011).

While no studies have reported that UCP2 directly affects δ-cells, PP-cells, and ε-cells, UCP2 may be involved in regulating the hormones secreted by these pancreatic islet cells (somatostatin, pancreatic polypeptide, and ghrelin, respectively). The modulation of these hormone levels is primarily influenced by blood glucose levels, with the balance of insulin and glucagon acting as the key regulatory mechanism (Lewandowski et al. 2024; Hoffman et al. 2023; Arafat et al. 2013). The direct role of the UCP2 gene in regulating somatostatin, pancreatic polypeptide, and ghrelin remains unclear. However, it is hypothesized that UCP2 may influence the secretion of these hormones by modulating the metabolic state and ROS levels within δ-cells, PP-cells, and ε-cells of the pancreatic islets (Coskun et al. 2013). Somatostatin plays a crucial role in inhibiting the release of other hormones such as insulin and glucagon (Henquin et al. 2017). The primary function of pancreatic polypeptide is to regulate pancreatic secretion and intestinal activity, while ghrelin primarily promotes appetite. Additionally, UCP2 can influence the release and regulation of pancreatic hormone levels by modulating gut hormone gastric inhibitory polypeptide (GIP) and pituitary adenylate cyclase-activating polypeptide (PACAP) (Zhou et al. 2005; Nakata et al. 2010). These hormones interact to form a complex regulatory network that sustains various biological functions in the body (Brink 2003; Röder et al. 2016; Müller et al. 2017). Collectively, UCP2 is a crucial target for pancreatic growth, development, and the maintenance of normal physiological function.

### Acute pancreatitis

AP arises primarily from the abnormal activation of pancreatic enzymes within acinar cells, initiating an inflammatory response and amplifying oxidative stress in a cascade effect. This process induces cell death, aggravates tissue damage, and can progress to systemic inflammatory response syndrome (SIRS), making it a life-threatening acute abdominal condition (He et al. 2024). UCP2 has been implicated in the pathophysiology of AP and its more severe form, SAP (Müller et al. 2014). This involvement occurs through various mechanisms, primarily due to the function of UCP2 in regulating mitochondrial function and modulating oxidative stress (Geng et al. 2024). In AP, mitochondrial dysfunction and oxidative stress are key factors in cellular injury. By regulating mitochondrial membrane potential and ROS levels, UCP2 may help maintain mitochondrial integrity and function, thereby reducing the severity of mitochondrial injury during AP (Hu et al. 2023).

Significantly higher transcript levels of UCP2 were observed in two classic animal models of pancreatitis—the continuous cerulein-injected mouse model and the taurocholic acid-injected rat model—compared to the control group (Segersvärd et al. 2005). The high expression of UCP2 suggested increased pancreatic follicular cell damage and a higher degree of pancreatitis. Interestingly, a greater degree of pancreatitis due to UCP2 knockout was observed only in aged UCP2-deficient mice (12 months old) and was more pronounced in the late stages of pancreatitis induction by sequential cerulein injections (24 h and 7 days after AP) (Segersvärd et al. 2005). In contrast, the degree of AP inflammation induced by UCP2 knockout in young mice did not differ from that in wild-type mice. Moreover, pancreatic enzymes were not significantly activated in UCP2 knockout pancreatitis mice, suggesting that the onset of pancreatitis in aged UCP2 knockout mice is not significantly related to pancreatic acinar cell activation (Müller et al. 2014).

Based on this, primary pancreatic stellate cells (PSCs) were extracted from aged UCP2 knockout mice and wild-type (WT) mice for further study. The results showed that the proliferation rate of PSCs from UCP2 knockout mice was lower than that of WT mice. However, there were no significant differences in aging rate, ROS levels, fat droplet loss, or fibrosis degree compared to the corresponding WT cells (Muller et al. 2016). These findings suggest that UCP2 knockout delays pancreatic repair by affecting PSCs proliferation. Persistent activation of PSCs is the main cause of CP (Wang et al. 2023), indicating that targeting UCP2 may have significant translational potential for its diagnosis and treatment (Yang et al. 2022a). Currently, there are no studies on the role of UCP2 in CP. However, our team has conducted in-depth research in this area and discovered some interesting findings, which we will report in due course.

Additionally, studies have confirmed that UCP2 knockout counteracts the inhibitory effects of marine on SAP-induced lung injury and ferroptosis, highlighting the important role of UCP2 in SAP progression (Jin et al. 2023). Low SIRT1 expression decreases intracellular NAD^+^ levels and inhibits the deacetylation of critical downstream molecules, promoting the development and progression of AP (Shen et al. 2017). Targeting SIRT1 has shown promise as an effective strategy to suppress AP progression. (Wang et al. 2021a; Bansod and Godugu 2021; Abdelzaher et al. 2021) Additionally, obesity—an escalating global health challenge—is linked to a rising incidence of obesity-related AP. PGC-1α plays a pivotal role in obesity-related AP; in obese states, pancreatic PGC-1α levels are suppressed, which prevents its binding to the NF-κB subunit p65, thereby promoting oxidative damage and amplifying IL-6-mediated inflammation, worsening AP severity (Pérez et al. 2019). Importantly, SIRT1, PPARγ, PGC-1α, and UCP2 constitute an interconnected regulatory network that jointly governs cellular energy metabolism, oxidative stress response, and inflammation (Oberkofler et al. 2009) SIRT1 modulates the activities of PGC-1α and PPARγ, both of which subsequently influence UCP2 expression levels, helping cells maintain stability and an anti-inflammatory state during metabolic stress or disease conditions. This regulatory interplay among these factors plays a crucial role in the pathogenesis of AP. In conclusion, UCP2 may be an important therapeutic target for pancreatitis and a key focus for future research.

### Pancreatic endocrine diseases

Endocrine diseases of the pancreas involve disorders of the hormone-producing cells in the pancreas. These primarily include Diabetes Mellitus, Insulinoma, Gastrinoma, Glucagonoma, Multiple Endocrine Neoplasia Type 1 (MEN1), Somatostatinoma, VIPoma, and Congenital Hyperinsulinism (CHI).

Diabetes Mellitus, the most common endocrine disease of the pancreas, is categorized into Type 1 and Type 2. Type 1 diabetes results from the autoimmune destruction of pancreatic beta-cells, leading to insufficient insulin secretion. In contrast, Type 2 diabetes is characterized by insulin resistance and inadequate insulin secretion. Table 2 summarizes studies related to UCP2 in pancreatic endocrine diseases, particularly diabetes mellitus, highlighting its role in glucose metabolism, insulin secretion, and oxidative stress. These studies collectively suggest that UCP2 plays a significant role in the pathogenesis of pancreatic endocrine diseases (González-Barroso et al. 2008; Gomathi et al. 2019; Mizusawa et al. 2022; Inoue et al. 2022; Giri et al. 2022; Yang et al. 2022b; Liu et al. 2022, 2014; Grubelnik et al. 2022; Buckels et al. 2021; Li et al. 2021; Odei-Addo et al. 2021; Naderi et al. 2020; Tavoosi et al. 2020; Sankaranarayanan and Kalaivani 2020; Yoo et al. 2020; Wade et al. 2019; Plecitá-Hlavatá et al. 2019; Wang et al. 2019; Maiztegui et al. 2018; Demirbilek and Hussain 2017; Matsunaga et al. 2014; Hals et al. 2012; Han et al. 2004).

**Table 2:** The studies related to UCP2 in pancreatic endocrine diseases

Years	Type	UCP2 level	Main findings	Refs.
2021	Congenital Hyperinsulinism	Down-regulation	UCP2, as one of the 16 key genes, is involved in regulating insulin secretion by pancreatic β-cells	Giri et al. (2022)
2022	Type 2 diabetes	Up-regulation	All-trans retinoic acid modulates the RXR/SREBP-1c/UCP2 signaling axis, thereby inhibiting insulin secretion and promoting the progression of diabetes	Yang et al. (2022b)
2022	pancreatic islet after severe burns	Up-regulation	Nicotinamide mononucleotide could maintain mitochondrial function through the SIRT1-UCP2 axis	Liu et al. (2022)
2022	Prss53 knockdown murine MIN6 β-cells	Up-regulation	The inhibition of UCP2 by mitochondrial Prss53 plays an auxiliary role in maintaining beta cell health	Mizusawa et al. (2022)
2022	Type 2 diabetes	Up-regulation	UCP2 upregulation is associated with β-cell failure, and the UCP2/AldB axis is a potential target for restoring β-cell function	Inoue et al. (2022)
2022	Pre- type 2diabetic hyperlipidemia	Up-regulation	Chronic high levels of free fatty acids upregulate UCP2, leading to β-cell dysfunction. This dysfunction is characterized by β-cells remaining highly active during hypoglycemia but becoming functionally quiescent during hyperglycemia	Grubelnik et al. (2022)
2021	Fetal growth restriction	Up-regulation	UCP2 may mediate IGF-I in a sex-specific manner to alter pancreatic endocrine function in adult children with fetal growth restriction	Buckels et al. (2021)
2021	Chronic adrenergic-stimulated beta cells	Down-regulation	Persistently low levels of UCP2 mediate the long-term adaptation of beta cells to adrenergic signaling	Li et al. (2021)
2021	Type 2 diabetes	Adipose tissue upregulated, liver tissue downregulated	High expression of UCP2 in adipose tissue may mediate the inhibitory effects of Leonurus extract and marrubium on type 2 diabetes	Odei-Addo et al. (2021)
2021	STZ-induced type 1 diabetic rats	Up-regulation	The effects of Tropisetron in type 1 diabetes are associated with modulation of the UCP2/ZnT8 signaling pathway and amelioration of oxidative stress	Naderi et al. (2020)
2020	Type 1 diabetes cell model	Down-regulation	Protective effects of cerium and yttrium oxide nitrogen oxides on CRI-D2 β cell lines exposed to H_2_O_2_ are associated with the regulation of UCP2	Tavoosi et al. (2020)
2020	HFD/STZ-induced type 2 diabetic rats	Up-regulation	Down-regulation of UCP2 expression by isoproterenol attenuates oxidative and ER stress responses in high-fat combined with STZ-induced diabetic rats	Sankaranarayanan and Kalaivani (2020)
2020	Type 2diabetes cell model	Up-regulation	Chebulic acid downregulates UCP2 to prevent MG-induced development of insulin sensitivity and oxidative stress-induced β-cell dysfunction	Yoo et al. (2020)
2019	Diabetes	Up-regulation	RNF20 and RNF40 regulate β-cell gene expression and insulin secretion associated with the regulation of UCP2	Wade et al. (2019)
2019	Type 2diabetes cell model	/	UCP2 promotes an antioxidant mechanism based on SkQ1^+^ fatty acid anion pairing	Plecitá-Hlavatá et al. (2019)
2019	Type 2diabetes	Polymorphism	UCP2 polymorphism affects insulin secretion leading to type 2 diabetes mellitus	Gomathi et al. (2019)
2019	Type 2diabetes cell model	Up-regulation	RP3-SeNPs down-regulate UCP2 to exert anti-oxidative stress effects	Wang et al. (2019)
2018	Type 2diabetes	Up-regulation	Upregulation of UCP2 affects pancreatic β-cell function	Maiztegui et al. (2018)
2017	Hyperinsulinaemic hypoglycaemia	/	UCP2 mutations affect the regulation of insulin secretion in pancreatic β-cells as a potential molecular mechanism leading to Hyperinsulinaemic hypoglycemia	Demirbilek and Hussain (2017)
2014	Type 2diabetes	Down-regulation	Up-regulation of UCP2 expression after berberine treatment is an important mechanism of its antidiabetic action	Liu et al. (2014)
2014	Chronic high glucose	Down-regulation	Glucotoxicity leading to beta-cell hypoxia is associated with down-regulation of UCP2	Matsunaga et al. (2014)
2013	Alpha cell-specific UCP2 knockout mice	Down-regulation	UCP2 is an essential gene for glucose sensing and maintenance of normal function in normal alpha cells	Allister et al. (2013)
2012	Type 2diabetes	Up-regulation	Effects on mitochondrial metabolism were possible only after a fourfold increase in UCP2 expression levels	Hals et al. (2012)
2011	βcell-specific UCP2 knockout mice	Up-regulation	UCP2 regulates ROS levels more significantly in β-cells	Robson-Doucette et al. (2011)
2009	Type 2diabetes	Down-regulation	UCP2 inhibition leads to enhanced insulin secretion and impaired α-cell function	Lee et al. (2009)
2008	Congenital Hyperinsulinism	Down-regulation	UCP2 knockout affects mitochondrial function and insulin secretion leading to hyperinsulinemic hypoglycemia	González-Barroso et al. (2008)
2007	Autoimmune diabetes	Down-regulation	Ucp2-KO mouse model of autoimmune diabetes has more severe symptoms	Emre et al. (2007)
2004	Type 2diabetes	Up-regulation	Inhibition of glucose sensitivity by taurine in UCP2 overexpressing β-cells was associated with an increased ATP/ADP ratio	Han et al. (2004)

Although the exact role of high and low UCP2 expression levels in these diseases is controversial, most studies indicate that increased UCP2 expression is generally associated with impaired insulin secretion and reduced β-cell function, contributing to hyperglycemia. Glucose-stimulated insulin secretion (GSIS) is essential for the endocrine regulation of the pancreas (Seshadri et al. 2017). Impaired GSIS is a significant contributor to insulin resistance and β-cell failure in type 2 diabetes mellitus. Furthermore, the upregulation of UCP2 is believed to be a contributing factor to impaired GSIS (Affourtit et al. 2011; Brand et al. 2010). Additionally, UCP2 influences mitochondrial function and reactive oxygen species production, further impacting cellular metabolism and insulin resistance. The main evidence supporting this view includes: (1) increased insulin secretion in UCP2 knockout mice (Zhang et al. 2001; Patanè et al. 2002), (2) elevated UCP2 expression levels strongly associated with high blood glucose levels (Brown et al. 2002), and (3) the therapeutic effect observed upon UCP2 knockout in mice modeling Type 2 diabetes (Zhang et al. 2001). However, contrary results were observed in another in vivo study (Pi et al. 2009), where UCP2 overexpression showed conflicting findings, with inhibitory, promotional, or no effects on β-cell function (Hong et al. 2001; Produit-Zengaffinen et al. 2007; Wang et al. 1999).

To verify this paradoxical phenomenon, Ingrid K. et al. (Hals et al. 2012). elevated UCP2 expression levels in β-cells in vitro to assess effects on parameters related to mitochondrial metabolism, including cell viability, apoptosis, insulin secretion, glucose oxidation, glutamine metabolism, mitochondrial membrane potential, mitochondrial mass, mitochondrial uncoupling, and ROS levels. Their results indicated that effects on β-cell metabolic levels were observed only when UCP2 levels were elevated more than four-fold. This study suggests that the role of UCP2 in blood glucose regulation and diabetes may not be concentration-dependent. Instead, a complex regulatory network centered on UCP2 likely exists, where high UCP2 levels may exert a protective effect in the pre-diabetic phase, but inhibit β-cell function under prolonged hyperglycemia. Since this study was conducted only in vitro and did not evaluate the effect of UCP2 expression levels on pancreatic islets, it may not fully elucidate the exact role of UCP2, but it is worthwhile to pursue further investigation.

### Pancreatic cancer

Pancreatic cancer encompasses a group of malignant tumors primarily arising from the pancreatic ductal epithelium and follicular cells. It is characterized by an insidious onset, challenging early diagnosis, rapid progression, short survival time, and poor prognosis (He et al. 2020). Pancreatic ductal adenocarcinoma (PDAC), the most prevalent pathological type, accounts for over 90% of cases (Wang et al. 2021b). The metabolic profile of PDAC is unique and complex, reflecting a high degree of metabolic flexibility to meet its growth and survival needs. The metabolic reprogramming features of PDAC include the Warburg effect, glutamine dependence, alterations in cholesterol and fatty acid metabolism, and resistance to oxidative stress (Santis et al. 2024). The significant reliance of pancreatic cancer on mitochondrial metabolism can lead to oxidative phosphorylation to produce ATP, driving malignant phenotypes such as metastasis and treatment resistance. Therefore, targeting mitochondrial metabolism is a promising therapeutic approach for pancreatic cancer. However, specifically targeting mitochondria without off-target effects in normal tissues remains a significant challenge (Yin et al. 2022).

Although precise targeting of mitochondrial function is still a distant goal, oxidative phosphorylation regulated by these organelles is indispensable in the metabolic reprogramming of PDAC. Specifically, the metabolic homeostasis of glutamine and aspartate is critical in this process (Caggiano and Taniguchi 2024). However, the key molecules involved in these energy metabolic pathways in PDAC tumorigenesis and progression cannot traverse the mitochondria alone; they require carriers to transport them to the inner mitochondrial membrane. Thus, UCP2, a member of the SLC25 family acting as a transmembrane anion carrier, may play a role in PDAC progression (Li et al. 2013). Numerous studies have detailed how UCP2 regulates glutamine and aspartate metabolism, particularly its role in mitochondrial energy regulation via the tricarboxylic acid (TCA) cycle and ROS management (Caggiano and Taniguchi 2024; Lauria et al. 2023). KRAS mutations, the most prevalent mutations in PDAC, impact not only cancer cells but also the tumor microenvironment. These mutations promote the tumor mesenchymal response and angiogenesis by secreting various cytokines and growth factors, thus creating a more favorable growth environment for tumor cells (Buscail et al. 2020). Notably, recent research indicates that UCP2-mediated aspartate transport is a crucial step in KRAS-regulated glutamine metabolism (Raho et al. 2020).

It is widely recognized that UCP2 expression is upregulated in PDAC (Caggiano and Taniguchi 2024). Table 3 summarizes studies related to UCP2 in Pancreatic cancer. UCP2 is downregulated before the tumor is fully formed to promote ROS accumulation and genomic instability (Lauria et al. 2023). In the later stages of tumorigenesis, UCP2 expression levels are upregulated to meet the metabolic needs of the tumor tissue, such as maintaining high ATP production, providing ROS protection, promoting therapeutic resistance, and facilitating immune evasion (Donadelli et al. 2015). Collectively, these results demonstrate the specificity and significance of UCP2 in PDAC progression, suggesting that UCP2 could serve as a potential therapeutic target for PDAC.

**Table 3:** Studies related to UCP2 in Pancreatic cancer

Years	Type	UCP2 role	Signaling pathway	Potential value	Refs.
2012	PDAC cell lines PaCa44, PaCa3, Panc1, CFPAC1, T3M4, and MiaPaCa2	Mitochondrial uncoupling of UCP2 mediates the mechanism of PDAC resistance to gemcitabine	ROS-mediated apoptosis pathway	UCP2 mediates PDAC gemcitabine chemotherapy drug resistance	Dalla et al. (2012)
2015	PDAC cell lines Panc1 and PaCa44	Onconase induces mitochondrial ROS production by inhibiting UCP2 expression levels	ROS/Akt/mTOR axis	UCP2 mediates the chemosensitivity of PDAC to gemcitabine	Fiorini et al. (2015)
2016	PDAC cell lines Panc-1	UCP2 inhibits ROS levels to induce pancreatic cancer cell death	ROS-mediated apoptosis pathway	UCP2 as a potential target for pancreatic cancer therapy	Yang et al. (2016)
2016	PDAC cell lines PaCa44 and Panc1	UCP2 mediates the metabolic transition of PDAC from mitochondrial oxidative phosphorylation to glycolysis	Induction of hnRNPA2/B1 and stimulation of GLUT1, PKM2 expression and L-lactate secretion	Inhibition of the UCP2-mediated glycolytic pathway promises a new approach to cancer therapy	Brandi et al. (2016)
2017	PDAC cell lines PaCa44, PaCa3, Panc1, MiaPaCa2, and T3M4	UCP2 overexpression promotes chemoresistance in PDAC	ROS/Akt/mTOR axis and GAPDH nuclear translocation	Combined inhibition of UCP2 and Akt/mTOR pathways is a novel therapeutic strategy for pancreatic cancer	Dando et al. (2017)
2020	PDAC cell lines PANC–1, SW1990, CAPAN–2, CFPAC–1, PANC0403, and BxPC–3	The HOTTIP-HOXA13 pathway promotes PDAC progression by upregulating UCP2 expression levels	HOTTIP–HOXA13 axis and HOTTIP–WDR5–MLL1–H3K4me3 pathway	Targeting downstream effector molecules of the HOTTIP pathway, including UCP2, could lead to the development of new PDAC therapies	Wong et al. (2020)
2020	PDAC cell lines Patu8988T, Panc1 and BxPC3	UCP2 connects the mitochondrial and cytoplasmic responses required for KRAS in PDAC rewired for glutamine metabolism	UCP2/ROS axis	UCP2 is a key metabolic target for the treatment of refractory tumors like PDAC	Raho et al. (2020)
2023	Murine cell line 6606PDA and Panc02	Ucp2 regulates the tumor microenvironment in favor of PDAC progression	Tumor stroma-related pathways	UCP2 promises to be a therapeutic target for PDAC	Revskij et al. (2023)

### UCP2-regulated macrophage phenotypic transformation in the pathogenesis of pancreatic diseases

Macrophages, a type of immune cell within the pancreatic microenvironment, play a pivotal role in the progression and pathogenesis of AP, CP, and pancreatic cancer (Wu et al. 2020). Their phenotypic transformation primarily involves macrophage polarization and macrophage-to-myofibroblast transition (MMT). Traditionally, M1 macrophage polarization is considered a key driver in the progression of AP and SAP (Peng et al. 2023), while M2 macrophage polarization, which exerts anti-inflammatory and pro-fibrotic effects, contributes to fibrosis in CP (Xue et al. 2015). In pancreatic cancer, M2 macrophages primarily mediate tissue repair and immune suppression, thereby promoting a microenvironment conducive to tumor progression (He et al. 2022). UCP2 is notably involved in the regulation of macrophage function, particularly in macrophage polarization. Studies indicate that UCP2 modulates the polarization of human primary macrophages (Lang et al. 2023). In AP, especially in obesity-associated AP, FABP4 upregulates UCP2, which in turn reduces oxidative stress to modulate macrophage signaling and inflammatory responses (Dierendonck et al. 2020; Steen et al. 2017). UCP2-regulated mitochondrial respiration acts as a crucial regulatory mechanism for IL-33-induced M2 macrophage polarization, facilitating the progression of CP (Faas et al. 2021). Additionally, macrophages are essential mediators in tissue repair following AP and contribute to the progression of pancreatic cancer (Wu et al. 2020). Furthermore, UCP2 regulation of macrophage-mediated NO/ROS damage is implicated in the progression of type 1 diabetes (Emre et al. 2007).

More recently, it has been discovered that certain macrophages can directly differentiate into myofibroblasts through a process known as MMT (Vierhout et al. 2021). While no studies to date have reported MMT in pancreatitis or pancreatic cancer, MMT is known to contribute to the progression of fibrotic diseases, such as kidney fibrosis, and cancers, including lung cancer (Wang et al. 2017; Tang et al. 2024). Indirect evidence suggests that STAT6-PPARα interactions regulate MMT, mediating kidney fibrosis progression (Yuan et al. 2023). This evidence supports the reasonable hypothesis that MMT may also play a role in pancreatic diseases, particularly in CP and pancreatic cancer, with UCP2 likely influencing this process to some extent. Overall, UCP2-regulated macrophage phenotypic transformation appears to significantly impact the progression of pancreatic diseases, lending further support to the hypothesis that UCP2 is a central regulatory factor in these conditions.

## Signaling pathways related to UCP2 regulation

Given the significant role of the UCP2 gene in regulating energy homeostasis, ROS, insulin secretion, and overall metabolism, as well as its critical regulatory role in pancreatic diseases.ROS generated by metabolic stress in the mitochondria of β-cells activates several ROS-related signaling pathways, such as the AMP-activated protein kinase (AMPK), Wnt, and nuclear factor kappa B (NF-κB) (Beall et al. 2013; Wang et al. 2014; Yu et al. 2020). These pathways, on the one hand, activate UCP2, causing proton leakage across the inner mitochondrial membrane and reducing ATP synthesis. On the other hand, they disrupt membrane integrity by oxidizing polyunsaturated fatty acids in the mitochondrial membrane, leading to the release of cytochrome c into the cytoplasm and inducing cellular apoptosis and autophagy (Ma et al. 2012; Dando et al. 2013). The regulatory relationship between UCP2 and ROS-related pathways not only influences pancreatic endocrine diseases by affecting the insulin secretory function of β-cells but also contributes to the progression of AP and PDAC.

The AMPK signaling pathway significantly affects UCP2 expression. During cellular energy stress, AMPK is activated to restore energy homeostasis and upregulate UCP2 expression by enhancing catabolism and inhibiting anabolism (Luo et al. 2022). Activated AMPK directly affects transcription factors like PPAR and SIRT1 to promote UCP2 transcription and enhances mitochondrial biogenesis by regulating coactivators like PGC-1α, further upregulating UCP2 (Xu et al. 2021b). AMPK activation also promotes fatty acid oxidation, regulates ROS levels, reduces oxidative stress and mitochondrial membrane potential, prevents oxidative damage, and maintains cellular function (Tripathi et al. 2023; Zhao et al. 2022). Additionally, AMPK influences glucose metabolism and insulin sensitivity (Entezari et al. 2022).

No studies have reported NF-κB binding to the κB site in the UCP2 gene promoter region. Like AMPK, NF-κB can regulate UCP2 expression in concert with coactivators (Wei et al. 2021). Inflammatory cytokines activate NF-κB, increasing UCP2 expression as part of the cellular response to inflammation and oxidative stress. UCP2 helps attenuate mitochondrial damage and maintain cellular homeostasis (Pan et al. 2021). NF-κB activation is often accompanied by elevated ROS levels. UCP2 reduces oxidative stress by lowering mitochondrial membrane potential and ROS production, providing feedback to control inflammation and oxidative damage (Adelakun et al. 2022). By regulating UCP2, NF-κB affects cellular energy metabolism. UCP2 uncouples oxidative phosphorylation, decreasing ATP production and increasing thermogenesis, impacting energy homeostasis during inflammation and stress responses (Zhang et al. 2020).

GSIS and the renin-angiotensin system (RAS) play crucial roles in pancreatic endocrinology. Palmitate-induced oxidative stress in β-cell mitochondria serves as a primary cellular model for GSIS impairment (Shaheen and Aljebali 2016), and several studies have shown that while UCP2 is not involved in palmitate-induced ROS generation, its upregulation protects against this damage (Li et al. 2017; Barlow et al. 2015; Hirschberg and Affourtit 2015). Blockade of RAS has been found to inhibit inflammation, oxidative stress in organelles, and apoptosis in pancreatic islet cells in a long-term high-fat diet rat model (Yuan et al. 2013). Accumulation of free fatty acids (FAs) induces oxidative stress, impairing pancreatic β-cell function (Ježek et al. 2015), with more pronounced damage from polyunsaturated FAs and their lipid peroxidation products compared to saturated FAs and their metabolites, possibly due to more extensive regulatory pathways mediating proton leakage, ATP synthesis, and ROS generation (Beck et al. 2007; Hu et al. 2017). Sustained ROS stimulation has been shown to directly damage β-cells by upregulating the JNK/P38 signaling pathway and activating UCP2 (Bo et al. 2016), with the glutathionylated state of UCP2 contributing to the regulation of GSIS levels in pancreatic islet cells (Mailloux et al. 2012). Collectively, UCP2 plays a crucial role in regulating energy homeostasis, ROS, insulin secretion, and overall metabolism, influencing the progression of pancreatic diseases and β-cell function via pathways including AMPK, Wnt, and NF-κB.

## Prospects and challenges

Consumption of foods rich in long-chain fatty acids, such as black soybeans and raw donkey's milk, has been shown to modestly increase UCP2 expression, potentially mitigating oxidative stress-related diseases. (Lionetti et al. 2012; Kanamoto et al. 2011) This offers a potential preventive strategy against the progression from AP, PDAC, and CP to pancreatic cancer. Earlier, we discussed the regulation of UCP2 via the AMPK signaling pathway (Beall et al. 2013). Metformin, a classic drug for type 2 diabetes, exerts hypoglycemic effects by activating the AMPK-mediated catabolic pathway, influencing blood glucose levels. Recently, its therapeutic potential in pancreatic cancer and other inflammatory conditions has gained considerable attention (Xu et al. 2022; Eibl and Rozengurt 2021; Gong et al. 2014). Therefore, metformin and other AMPK modulators show promise in pancreatic diseases and warrant further investigation as potential novel therapies. Additionally, traditional Chinese medicine, with its millennia-long foundation, also exhibits regulatory effects on UCP2 (Sun et al. 2024; Yang et al. 2011). Combining UCP2 with chemotherapeutic agents as an adjuvant strategy shows potential application value, enhancing effectiveness in inhibiting pancreatic cancer (Dalla et al. 2012; Fiorini et al. 2015). In conclusion, while the theoretical foundation supports the potential application of UCP2 in pancreatic diseases, clinical validation is necessary.

Inevitably, there are challenges for UCP2 as a therapeutic target for pancreatic diseases. Firstly, tissue specificity and selectivity pose significant challenges, as UCP2 is widely distributed across various tissues, making it difficult to design inhibitors or activators that are highly specific to pancreatic tissue. Secondly, systemic modulation of UCP2 may cause side effects, given its diverse roles in different tissues. For instance, excessive inhibition of UCP2 could lead to abnormal energy metabolism and dysfunction in other tissues. Additionally, the precise mechanisms of UCP2's action in pancreatic diseases remain inadequately understood. UCP2's multiple roles in energy metabolism, oxidative stress, apoptosis, and immune responses complicate targeting strategies, preventing the focus on a single specific role. Finally, extensive studies and validations are required to determine the efficacy and safety of UCP2-targeted therapies, transitioning from basic research to clinical applications, and design rational clinical trial protocols.

## Conclusions

UCP2 is broadly expressed in numerous tissues, including the pancreas, and demonstrates the highest homology between humans and mice. UCP2 is involved in various physiological functions, such as cellular energy metabolism, oxidative stress management, insulin secretion, lipid regulation, metabolic reprogramming, and immune modulation. UCP2 plays a role in regulating both endocrine and exocrine pancreatic functions. Epidemiological data on pancreatic diseases, such as acute AP, CP, pancreatic cancer, and diabetes, indicate concerning trends, with evidence suggesting frequent interconversion among these conditions. However, the understanding of these diseases’ pathogenesis and interrelationships remains limited, particularly in identifying and validating key molecules that may connect or transform these conditions. UCP2 is expected to serve as such a key target. This review presents a comprehensive analysis of current research on UCP2’s role in pancreatic diseases. We discuss recent findings on UCP2’s complex regulatory mechanisms, propose UCP2 as a central regulatory factor in pancreatic disease progression, and hypothesize that UCP2 dysfunction could significantly contribute to disease pathogenesis and interconversion. Clarifying UCP2’s role and mechanisms in pancreatic diseases could provide new directions for therapeutic and diagnostic innovation.

## Data Availability

No datasets were generated or analysed during the current study.

## Abbreviations

UCP2: Mitochondrial uncoupling protein 2
MIPS: Mitochondrial information processing system
UCPs: Uncoupling proteins
ROS: Reactive oxygen species
PACAP: Pituitary adenylate cyclase-activating polypeptide
GIP: Gastric inhibitory polypeptide
AP: Acute pancreatitis
SAP: Severe acute pancreatitis
PSCs: Pancreatic stellate cells
WT: Wild-type
CP: Chronic pancreatitis
MEN1: Multiple Endocrine Neoplasia Type 1
CHI: Congenital hyperinsulinism
GSIS: Glucose-stimulated insulin secretion
PDAC: Pancreatic ductal adenocarcinoma
TCA: Tricarboxylic acid
SNPs: Single nucleotide polymorphisms
Indels: Insertion/deletion polymorphisms
RSPs: Repetitive sequence polymorphisms
SV: Structural variants
Foxa1: Forkhead box protein O1
SIRT1: Silent mating type information regulation 2 homolog-1
SREBP: Sterol regulatory element binding protein isoforms
TRE: Thyroid hormone response elements
E-box: Helix-loop-helix protein binding sites
PGC-1α: Peroxisome proliferator-activated receptor-γ coactivator-1 α
PPARs: Peroxisome proliferator-activated receptors
RXR: Retinoid X-like receptor
Uorf: Upstream open reading frame
NcRNAs: Non-coding RNAs
miRNAs: MicroRNAs
LncRNAs: Long non-coding RNAs
AMPK: AMP-activated protein kinase
NF-κB: Nuclear factor kappa B
RAS: Renin-angiotensin system
FAs: Fatty acids
MMT: Macrophage-to-myofibroblast transition
SIRS: Systemic inflammatory response syndrome
